# clustermq enables efficient parallelization of genomic analyses

**DOI:** 10.1093/bioinformatics/btz284

**Published:** 2019-05-27

**Authors:** Michael Schubert

**Affiliations:** European Molecular Biology Laboratory, European Bioinformatics Institute (EMBL-EBI), Wellcome Trust Genome Campus, Cambridge, UK

## Abstract

**Motivation:**

High performance computing (HPC) clusters play a pivotal role in large-scale bioinformatics analysis and modeling. For the statistical computing language R, packages exist to enable a user to submit their analyses as jobs on HPC schedulers. However, these packages do not scale well to high numbers of tasks, and their processing overhead quickly becomes a prohibitive bottleneck.

**Results:**

Here we present *clustermq*, an R package that can process analyses up to three orders of magnitude faster than previously published alternatives. We show this for investigating genomic associations of drug sensitivity in cancer cell lines, but it can be applied to any kind of parallelizable workflow.

**Availability and implementation:**

The package is available on CRAN and https://github.com/mschubert/clustermq. Code for performance testing is available at https://github.com/mschubert/clustermq-performance.

**Supplementary information:**

Supplementary data are available at *Bioinformatics* online.

## 1 Introduction

The volume of data produced in the biological sciences has recently increased by orders of magnitude across many disciplines, most apparent in single cell sequencing (Svensson *et al.*, 2018). In order to analyze this data, there is a need not only for efficient algorithms, but also for efficient and user-friendly utilization of high performance computing (HPC). Having reached a limit in the speed of single processors, the focus has shifted to distributing computing power to multiple processors or indeed multiple machines. HPC clusters have played and are continuing to play an integral role in bioinformatic data analysis and modelling. However, efficient parallelization using low-level systems such as MPI, or submitting jobs that later communicate via network sockets, requires specialist knowledge. 

For the popular statistical computing language R (Ihaka and Gentleman, 1996) several packages have been developed that are able to automate parallel workflows on HPC without the need for low-level programming. The best-known packages for this are *BatchJobs* (Bischl *et al.*, 2015) and *batchtools* (Lang *et al.*, 2017). They provide a consistent interface for distributing tasks over multiple workers by automatically creating the files required for processing each individual computation, and collect the results back to the main session upon completion.

**Fig. 1.**
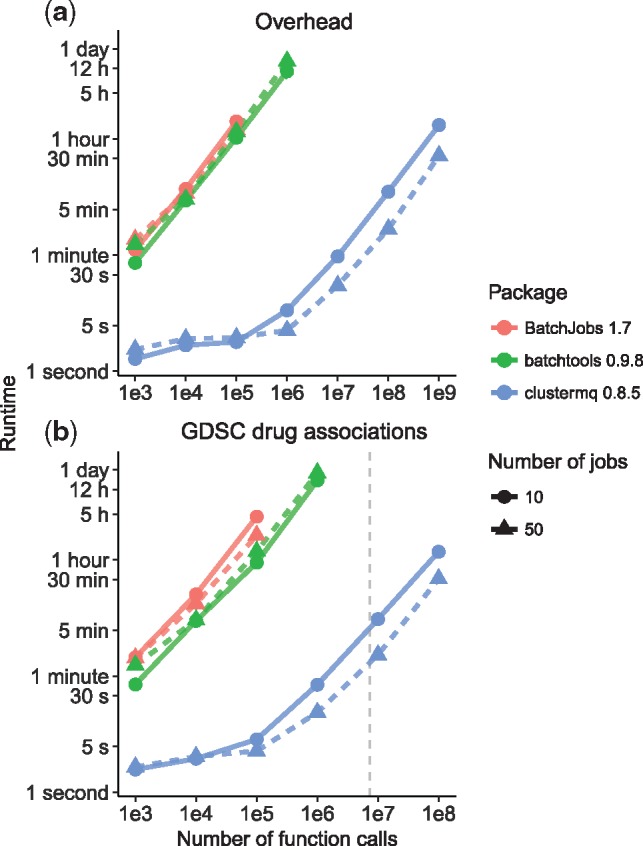
Performance evaluation of HPC packages for (**a**) processing overhead and (**b**) application to GDSC data. Along the range of tested number of function calls, *clustermq* requires substantially less time for processing in both scenarios. Indicated measurements are averages of two runs with range shown as vertical bars. (b) The dashed grey line indicates the actual number of calls required for all GDSC associations

However, these packages write arguments and results of individual function calls to a networked file system. This is highly inefficient for a large number of calls and effectively limits these packages at about 10^6^ function evaluations (cf. Fig. 1 and Supplementary Methods). In addition, it hinders load balancing between computing nodes (as it requires a file-system based lock mechanism) and the use of remote compute facilities without shared storage systems.

Here we present the R package *clustermq* that overcomes these limitations and provides a minimal interface to submit jobs on a range of different schedulers (LSF, SGE, Slurm, PBS, Torque). It distributes data over the network without involvement of network-mounted storage, monitors the progress of up to 10^9^ function evaluations and collects back the results.

## 2 Implementation

In order to provide efficient distribution of data as well as compute instructions, we use the *ZeroMQ* library (Hintjens, 2013), which provides a level of abstraction of simple network sockets and handles low-level operations such as message envelopes and timeouts. The main function used to distribute tasks on compute nodes and subsequently collect the results is the *Q* function. It takes named iterated arguments, and a list of *const* (objects that do not change their value between function calls) and export objects (which will be made available in the worker environment). The *Q* function will check which schedulers are available, and is hence often usable without any additional required setup (cf. Supplementary User Guide).

# load the library and create a simple function

library(clustermq)

fx= function(x, y) x * 2 + y

# queue the function call on your scheduler

Q(fx, x = 1: 3,const=list(y = 1),n_jobs = 1)

# list(3, 5, 7)

Another way of parallelization is to register *clustermq* as a parallel *foreach* backend. This is particularly useful if a third-party package uses *foreach* loops internally, like all *Bioconductor* (Gentleman *et al.*, 2004) packages that make use of *BiocParallel*.

library(foreach)

register_dopar_cmq(n_jobs = 2, memory = 1024)

# this will be executed as jobs

foreach(i = 1: 3)

# also for Bioconductor packages using this

BiocParallel:: register(BiocParallel::DoparParam())

BiocParallel::bplapply(1: 3,sqrt)

In addition, the package provides a documented worker API that can be used to build tools that need fine-grained control over the calls sent out instead of the normal scatter-gather approach (cf. Supplementary Technical Documentation).

## 3 Evaluation

In order to evaluate the performance of *clustermq* compared to the *BatchJobs* and *batchtools* packages (cf. Supplementary Methods), we first tested the overhead cost for each of these tools by evaluating a function of negligible runtime and repeating this between 1000 and 10^9^ times. We found that *clustermq* has about 1000× less overhead cost compared to the other packages when processing 10^5^ or more calls, although across the whole range a clear speedup is apparent (Fig. 1a). The maximum number of calls that *BatchJobs* could successfully process was 10^5^, while *batchtools* increased this limit to 10^6^. By contrast, *clustermq* was able to process 10^9^ calls in about one hour.

For our second evaluation, we chose a realistic scenario with application to biological data. The Genomics of Drug Sensitivity in Cancer (GDSC) project published molecular data of approximately 1000 cell lines and their response (IC_50_) to 265 drugs (Iorio *et al.*, 2016). We ask the question if any one of 1073 genomic or epigenomic events (mutation/copy number aberration of a gene and differential promoter methylation, respectively) is correlated with a significant difference in drug sensitivity across all cell lines or for 25 specific cancer types (*n* = 7 392 970 associations). We found that for this setup, *clustermq* is able to process the associations in about one hour with 10% lost to overhead (Fig. 1b; dashed line). The other packages produced too many small temporary files for our networked file system to handle, and by extrapolation processing all associations would have taken over a week.

To achieve similar results using the previously published packages one would need to adapt the analysis code to chunk together related associations and explicitly loop through different subsets of data. *clustermq* lifts this requirement and lets the analyst focus on the biological question they are trying to address instead of manually optimizing code parallelization for execution time (cf. Supplementary Discussion).

## 4 Conclusion

The *clustermq* R package enables computational analysts to efficiently distribute a large number of function calls via HPC schedulers, while reducing the need to adapt code between different systems. We have shown its utility for drug screening data, but it can be used a broad range of analyses. This includes *Bioconductor* packages that make use of *BiocParallel*. 

*Conflict of Interest*: none declared.

## Supplementary Material

btz284_Supplementary_Data
