# Supplementary material for: clustermq enables efficient parallelization of genomic analyses
Source: Bioinformatics. 2019 May 27;35(21):4493–5. doi: 10.1093/bioinformatics/btz284 (PMC6821287; doi:10.1093/bioinformatics/btz284)
Supplement: btz284_Supplementary_Data [file btz284_supplementary_data.pdf]

Supplementary Data for "clustermq enables efficient parallelisation of genomic analyses"

Michael Schubert

2019-04-11

Contents

Online methods **2**
Package architecture . . . 2
Testing hardware . . . 3
Package options . . . 3
Running test cases . . . 4
Overhead comparison . . . 4
GDSC comparison . . . 4

Discussion **4**

User guide **6**
Installation . . . 6
Configuration . . . 6
Usage . . . 7
Troubleshooting . . . 9
Environments . . . 10

Technical documentation **12**
Worker API . . . 12
ZeroMQ message specification . . . 14

Appendix: Scheduler templates **18**
LSF . . . 18
SGE . . . 19
SLURM . . . 20
PBS . . . 21
Torque . . . 22

Online methods

Package architecture

Traditionally, R packages that enable a user to distribute a set of function calls to workers on managed by a HPC scheduler rely on network-mounted storage (Fig. S1a). This means that for a certain set of calls, all arguments required for each of these needs to be written to disk. These files are then accessed by HPC workers after their jobs started up, which perform the computations. After computations are done, the workers again write their results on network storage.

This is not a problem if the total number of calls to process is low and run-times for each call themselves are high, as then the processing time will be determined by the task itself. However, if there is a high number of short-running calls to process, this becomes a serious bottleneck (cf. Fig. 1).

This bottleneck can be mitigated by processing multiple function calls in one chunk and reading/writing the whole chunk data from/to one file. This is, however, not what the commonly used tools `BatchJobs` and `batchtools` do. In testing different chunk sizes, we found that the number of result files scaled with the number of calls, not the number of chunks (cf. Table S1).

Table S1. Number of result files produced by `BatchJobs` and `batchtools`. Each function call produces one result file, irrespective of the number of chunks set to the number of workers or not.

Number of function calls	Files for BatchJobs	Files for batchtools
1e3	1,027	1,002
1e4	10,027	10,002
1e5	100,027	100,002

A user could still manually chunk together many short-running calls into fewer but longer calls (cf. discussion with the `batchtools` developers at https://github.com/mllg/batchtools/issues/222). Alternatively, the `future.apply` package could be used to do this automatically:

```r
library(future.apply)
plan(future.batchtools_sge, workers = 50)
y = future_lapply(1:10^9, FUN = sqrt)
```

The `clustermq` package on the other hand bypasses network storage entirely (Fig. S1b) and handles chunking automatically. It will transfer the common data required to perform any call to each worker once, and then with each call (or set of calls) transfer the iterated call arguments to the workers. This is not only faster as it does not incur the delay of both writing to and reading from a networked hard disk, but also allows for load balancing if a worker is faster or slower than others with its computations.

The network-based approach (Fig. S1b) has another advantage: If the R session is running on a personal computer that is connected to a computing cluster via SSH, computations can be sent to workers, and the results from the cluster returned to the local session without the need for additional setup (as long as R is installed with all relevant packages on the computing cluster as well as local session). In this case, `clustermq` transfers common objects to the computing cluster once, and then waits for the results using an SSH connection called reverse tunneling. For file-system-based tools on the other hand, a user would need to copy relevant data on the network-mounted storage of the computing cluster (or mount it locally via sshfs), submit jobs there manually, and copy/read the results afterwards back to the locally running session afterwards.

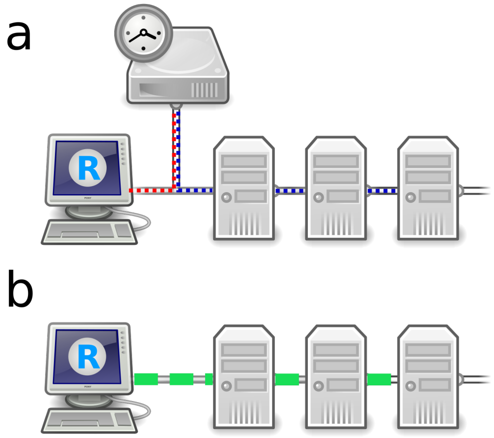

Fig. S1. Difference in design of (a) network storage-based parallelisation in `BatchJobs` and `batchtools` vs. (b) `clustermq`'s network-only solution. In the former, function arguments are written to disk for every call, then read by the workers, which in turn save their result on disk. This needs to be read by the main process again to collect the results. By contrast, `clustermq` distributes calls from memory, and collects results to memory without requiring additional disk storage.

Of note, workers need to be able to connect via the TCP/IP protocol to the master process in order for `clustermq` to work. These connections are authenticated using a session password passed as environment variable in the job submission script, but are not encrypted. Remote SSH sessions need support for reverse tunneling on the HPC login node, and are authenticated and encrypted via the SSH layer.

Testing hardware

We tested on a computing cluster with 157 nodes, each with 40 cores and 128 GB memory, using the LSF scheduler. It at each time had medium load and enough free slots to accommodate starting the measurements immediately (using 10 or 50 jobs).

Nodes were connected internally using a 10 Gbit ethernet connection. The network storage was a Lustre high performance file system.

Package options

`BatchJobs` calls were explicitly chunked with the number of chunks equal to the number of jobs. Completed jobs were reduced as a list. Queries were staged, the `SQLite` database timeout set to 5 seconds, and the journal mode to `WAL` (in line with recommendations to reduce issues with file-system locks). Removing the registry directory after job completion was counted as processing time.

For `batchtools` we used `btmapply` with default settings, except explicit chunking with the number of chunks equal to the number of jobs.

For `clustermq` we defined `rettype` as a numeric vector for the overhead scenario and as a list for the GDSC scenario. We reduced the number of calls to 10ˆ8 for the GDSC scenario in order to fit the results in the main task's memory.

Worker memory was set at 512 MB for all packages and all tests.

The number of calls was reduced for BatchJobs because of reproducible SQLite database errors for 10ˆ6 calls and over. It was also reduced for batchtools because the package did not successfully complete 10ˆ7 calls and over with our setup.

Running test cases

Every combination of package, number of calls, and number of jobs was run in two replicates. We randomized the order each of these measurements was taken. If jobs were queued long enough for this to influence the overall time it took for processing, the measurement was discarded and repeated.

We were running only one test case at any time. Before running each test case, we introduced a random delay between 30 and 60 seconds in order not to be penalized by the scheduler for rapid submissions.

Overhead comparison

The idea of a processing overhead comparison is that given negligible evaluation time of individual function calls, we can estimate how much time it takes a framework to distribute and process its calls, and collect the result after. This provides a lower bound at which a number of function calls can be processed. If the overhead cost of processing a number of function calls is too high by itself, it indicates that a certain framework is no longer suitable to process this number of tasks.

For our tests, we chose as input a vector of uniform random numbers of length N, and a function that multiplies each of these numbers by 2, resulting in N function calls. This ensures that we can verify the result by locally performing the same task. The time it takes to perform each of these multiplications is expected to be negligible compared to the time it takes to distribute each individual function call and collect the results. We chose N to be between 10^3 and 10^9 calls, separated by a factor of 10.

GDSC comparison

We downloaded the following files from the GDSC1000 web site (https://www.cancerrxgene.org/gdsc1000/GDSC1000_WebResources/Home.html):

- Annotated list of cell-lines
- log(IC50) values Cancer functional events (CFEs)
- BEMs for cell-lines

We filtered the data by cancer cohorts that had at least 10 cell lines, yielding 25 cohorts and 873 cell lines, their response to 265 drugs, and 1073 binary events that are either present or absent in a given cell line.

For each of these cohorts, we ran a linear model to test whether the drug sensitivity (IC50) is different for cell lines that harbor an event vs. those that do not. Additionally, we performed the same for the pan-cancer cohort. For each of these models, we converted the result into a data.frame using the broom package. This yielded in total of 7,392,970 associations.

As this is a fixed number that only gives limited insight into the processing capabilities of HPC packages, we resampled with replacement the combinations of cohort, drug, and binary event to yield a number between 10^3 and 10^8, separated by a factor of 10.

Discussion

While we observe a very large difference in processing times between `clustermq` and the other packages, it should be noted that both our test cases focussed on a high number of short-running function calls to be evaluated via HPC schedulers.

The difference in processing time we see can only in part be explained by the physical limitations of the network vs. the file system. A major contributor is that both `BatchJobs` and `batchtools` save the result of each function call in a separate results file irrespective of chunking, which causes additional latency that could be avoided by combining the results of a chunk in a single results file.

This issue has been raised with the authors of the `batchtools` package in their repository[1]. They have confirmed that this is a design choice for increased robustness: should a job's R process unexpectedly fail

[1] https://github.com/mllg/batchtools/issues/222

(e.g. due to a process segfault or hitting the job's wall time limit), all previously computed results should still be available. `clustermq` makes no such guarantees. It is the user's responsibility to reserve enough wall time, and computations that can not be completed will be lost.

Both `batchtools` as well as `clustermq` are perfectly capable of processing fewer and longer running function calls. Depending on the number/duration of calls and how precious every individual evaluation is, both have their use cases.

User guide

Installation

ZeroMQ

The `rzmq` package (which this package depends on) needs the system library ZeroMQ.

```
# You can skip this step on Windows and macOS, the rzmq binary has it
brew install zeromq # Linuxbrew, Homebrew on macOS
conda install zeromq # Conda
sudo apt-get install libzmq3-dev # Ubuntu
sudo yum install zeromq3-devel # Fedora
pacman -S zeromq # Arch Linux
```

More details can be found at the rzmq project README.

R package

The latest stable version is available on CRAN.

Alternatively, it is also available on the `master` branch of the repository.

```r
# from CRAN
install.packages('clustermq')

# from Github
# install.packages('devtools')
devtools::install_github('mschubert/clustermq')
```

In the `develop` branch, we will introduce code changes and new features. These may contain bugs, poor documentation, or other inconveniences. This branch may not install at times. However, feedback is very welcome.

```r
# install.packages('devtools')
devtools::install_github('mschubert/clustermq', ref="develop")
```

Configuration

Setting up the scheduler

An HPC cluster's scheduler ensures that computing jobs are distributed to available worker nodes. Hence, this is what `clustermq` interfaces with in order to do computations.

By default, we will take whichever scheduler we find and fall back on local processing. This will work in most, but not all cases.

To set up a scheduler explicitly, see the following links:

- LSF
- SGE
- Slurm
- Torque
- PBS
- if you want another scheduler, open an issue

SSH connector

There are reasons why you might prefer to not to work on the computing cluster directly but rather on your local machine instead. RStudio is an excellent local IDE, it's more responsive than and feature-rich than

browser-based solutions (RStudio server, Project Jupyter), and it avoids X forwarding issues when you want to look at plots you just made.

Using this setup, however, you lost access to the computing cluster. Instead, you had to copy your data there, and then submit individual scripts as jobs, aggregating the data in the end again. `clustermq` is trying to solve this by providing a transparent SSH interface.

In order to use `clustermq` from your local machine, the package needs to be installed on both there and on the computing cluster. On the computing cluster, set up your scheduler and make sure `clustermq` runs there without problems. On your local machine, add the following options in your `~/.Rprofile`:

```
options(
    clustermq.scheduler = "ssh",
    clustermq.ssh.host = "user@host", # use your user and host, obviously
    clustermq.ssh.log = "~/cmq_ssh.log" # log for easier debugging
)
```

We recommend that you set up SSH keys for password-less login.

Usage

The `Q` function

The following arguments are supported by `Q`:

- `fun` - The function to call. This needs to be self-sufficient (because it will not have access to the `master` environment)
- `...` - All iterated arguments passed to the function. If there is more than one, all of them need to be named
- `const` - A named list of non-iterated arguments passed to `fun`
- `export` - A named list of objects to export to the worker environment

Behavior can further be fine-tuned using the options below:

- `fail_on_error` - Whether to stop if one of the calls returns an error
- `seed` - A common seed that is combined with job number for reproducible results
- `memory` - Amount of memory to request for the job (`bsub -M`)
- `n_jobs` - Number of jobs to submit for all the function calls
- `job_size` - Number of function calls per job. If used in combination with `n_jobs` the latter will be overall limit
- `chunk_size` - How many calls a worker should process before reporting back to the master. Default: every worker will report back 100 times total

The full documentation is available by typing `?Q`.

Examples

The package is designed to distribute arbitrary function calls on HPC worker nodes. There are, however, a couple of caveats to observe as the R session running on a worker does not share your local memory.

The simplest example is to a function call that is completely self-sufficient, and there is one argument (`x`) that we iterate through:

```
fx = function(x) x * 2
Q(fx, x=1:3, n_jobs=1)
#> Submitting 1 worker jobs (ID: 6156) ...
#> [[1]]
#> [1] 2
#>
#> [[2]]
```

```
#> [1] 4
#>
#> [[3]]
#> [1] 6
```

Non-iterated arguments are supported by the `const` argument:

```
fx = function(x, y) x * 2 + y
Q(fx, x=1:3, const=list(y=10), n_jobs=1)
#> Submitting 1 worker jobs (ID: 6855) ...
#> [[1]]
#> [1] 12
#>
#> [[2]]
#> [1] 14
#>
#> [[3]]
#> [1] 16
```

If a function relies on objects in its environment that are not passed as arguments, they can be exported using the `export` argument:

```
fx = function(x) x * 2 + y
Q(fx, x=1:3, export=list(y=10), n_jobs=1)
#> Submitting 1 worker jobs (ID: 6855) ...
#> [[1]]
#> [1] 12
#>
#> [[2]]
#> [1] 14
#>
#> [[3]]
#> [1] 16
```

If we want to use a package function we need to load it on the worker using a `library()` call or referencing it with `package_name::`:

```
fx = function(x) {
    library(dplyr)
    x %>%
        mutate(area = Sepal.Length * Sepal.Width) %>%
        head()
}
Q(fx, x=list(iris), n_jobs=1)
#> Submitting 1 worker jobs (ID: 6855) ...
#>
#> Attaching package: 'dplyr'
#> The following objects are masked from 'package:stats':
#>
#>     filter, lag
#> The following objects are masked from 'package:base':
#>
#>     intersect, setdiff, setequal, union
#> [===========================================================] 100% eta: 0s
#> [[1]]
#>   Sepal.Length Sepal.Width Petal.Length Petal.Width Species  area
```

```
#> 1          5.1         3.5          1.4         0.2  setosa 17.85
#> 2          4.9         3.0          1.4         0.2  setosa 14.70
#> 3          4.7         3.2          1.3         0.2  setosa 15.04
#> 4          4.6         3.1          1.5         0.2  setosa 14.26
#> 5          5.0         3.6          1.4         0.2  setosa 18.00
#> 6          5.4         3.9          1.7         0.4  setosa 21.06
```

As parallel `foreach` backend

The `foreach` package provides an interface to perform repeated tasks on different backends. While it can perform the function of simple loops using `%do%`:

```
library(foreach)
x = foreach(i=1:3) %do% sqrt(i)
```

it can also perform these operations in parallel using `%dopar%`:

```
x = foreach(i=1:3) %dopar% sqrt(i)
#> Warning: executing %dopar% sequentially: no parallel backend registered
```

The latter allows registering different handlers for parallel execution, where we can use `clustermq`:

```
clustermq::register_dopar_cmq(n_jobs=2, memory=1024) # this accepts same arguments as `Q`
x = foreach(i=1:3) %dopar% sqrt(i) # this will be executed as jobs
#> Submitting 2 worker jobs (ID: 6665) ...
```

As BiocParallel supports `foreach` too, this means we can run all packages that use `BiocParallel` on the cluster as well via `DoparParam`.

With `drake`

The `drake` package enables users to define a dependency structure of different function calls, and only evaluate them if the underlying data changed.

> drake — or, Data Frames in R for Make — is a general-purpose workflow manager for data-driven tasks. It rebuilds intermediate data objects when their dependencies change, and it skips work when the results are already up to date. Not every runthrough starts from scratch, and completed workflows have tangible evidence of reproducibility. drake also supports scalability, parallel computing, and a smooth user experience when it comes to setting up, deploying, and maintaining data science projects.

It can use `clustermq` to perform calculations as jobs:

```
library(drake)
load_mtcars_example()
# clean(destroy = TRUE)
# options(clustermq.scheduler = "multicore")
make(my_plan, parallelism = "clustermq", jobs = 2, verbose = 4)
```

Troubleshooting

Debugging workers

Function calls evaluated by workers are wrapped in event handlers, which means that even if a call evaluation throws an error, this should be reported back to the main R session.

However, there are reasons why workers might crash, and in which case they can not report back. These include:

- A segfault in a low-level process
- Process kill due to resource constraints (e.g. walltime)
- Reaching the wait timeout without any signal from the master process
- Probably others

In this case, it is useful to have the worker(s) create a log file that will also include events that are not reported back. It can be requested using:

```r
Q(..., log_file="/path/to.file")
```

Note that `log_file` is a template field of your scheduler script, and hence needs to be present there in order for this to work. The default templates all have this field included.

In order to log each worker separately, some schedulers support wildcards in their log file names. For instance:

- LSF: `log_file="/path/to.file.%I"`
- Slurm: `log_file="/path/to.file.%a"`

Your scheduler documentation will have more details about the available options.

When reporting a bug that includes worker crashes, please always include a log file.

SSH

Before trying remote schedulers via SSH, make sure that the scheduler works when you first connect to the cluster and run a job from there.

If the terminal is stuck at

```
Connecting <user@host> via SSH ...
```

make sure that each step of your SSH connection works by typing the following commands in your **local** terminal and make sure that you don't get errors or warnings in each step:

```sh
# test your ssh login that you set up in ~/.ssh/config
# if this fails you have not set up SSH correctly
ssh <user@host>

# test port forwarding from 54709 remote to 6687 local (ports are random)
# if the fails you will not be able to use clustermq via SSH
ssh -R 54709:localhost:6687 <user@host> R --vanilla
```

If you get an `Command not found: R` error, make sure your `$PATH` is set up correctly in your `~/.bash_profile` and/or your `~/.bashrc` (depending on your cluster config you might need either).

If you get a SSH warning or error try again with `ssh -v` to enable verbose output.

If the forward itself works, set the following option in your `~/.Rprofile`:

```r
options(clustermq.ssh.log = "~/ssh_proxy.log")
```

This will create a log file *on the remote server* that will contain any errors that might have occurred during `ssh_proxy` startup.

Environments

In some cases, it may be necessary to activate a specific computing environment on the scheduler jobs prior to starting up the worker. This can be, for instance, because *R* was only installed in a specific environment or container.

Examples for such environments or containers are:

- Bash module environments

- Conda environments
- Docker/Singularity containers

It should be possible to activate them in the job submission script (i.e., the template file). This is widely untested, but would look the following for the LSF scheduler (analogous for others):

```
#BSUB-J {{ job_name }}[1-{{ n_jobs }}]  # name of the job / array jobs
#BSUB-o {{ log_file | /dev/null }}      # stdout + stderr
#BSUB-M {{ memory | 4096 }}             # Memory requirements in Mbytes
#BSUB-R rusage[mem={{ memory | 4096 }}] # Memory requirements in Mbytes
##BSUB-q default                        # name of the queue (uncomment)
##BSUB-W {{ walltime | 6:00 }}          # walltime (uncomment)

module load {{ bashenv | default_bash_env }}
# or: source activate {{ conda | default_conda_env_name }}
# or: your environment activation command
ulimit -v $(( 1024 * {{ memory | 4096 }} ))
R --no-save --no-restore -e 'clustermq:::worker("{{ master }}")'
```

This template still needs to be filled, so in the above example you need to pass either

```
Q(..., template=list(bashenv="my environment name"))
```

or set it via an *.Rprofile* option:

```
options(
    clustermq.defaults = list(bashenv="my default env")
)
```

Technical documentation

Worker API

Base API and schedulers

The main worker functions are wrapped in an `R6` class with the name of `QSys`. This provides a standardized API to the lower-level messages that are sent via `rzmq`.

The base class itself is derived in scheduler classes that add the required functions for submitting and cleaning up jobs:

```
+ QSys
  |- Multicore
  |- LSF
  + SGE
    |- PBS
    |- Torque
  |- etc.
```

A pool of workers can be created using the `workers()` function, which instantiates an object of the corresponding `QSys`-derived scheduler class. See `?workers` for details.

```r
# start up a pool of three workers using the default scheduler
w = workers(n_jobs=3)

# if we make an unclean exit for whatever reason, clean up the jobs
on.exit(w$finalize())
```

Worker startup

For workers that are started up via a scheduler, we do not know which machine they will run on. This is why we start up every worker with a TCP/IP address of the master socket that will distribute work.

This is achieved by the call to R common to all schedulers:

```sh
R --no-save --no-restore -e 'clustermq:::worker("{{ master }}")'
```

On the master's side, we wait until a worker connects:

```r
# this will block until a worker is ready
msg = w$receive_data()
```

Common data and exports

Workers will start up without any knowledge of what they should process or how. In order to transfer initial data to the worker, we first create and serialize a list object with the following fields:

- `fun` - the function to call with iterated data
- `const` - the constant data each function call should receive
- `export` - objects that will be exported to the workers' `.GlobalEnv`
- `rettype` - character string which data type to return; e.g. `list`, `logical`
- `common_seed` - random seed for function calls; will be offset by job ID
- `token` - character string to identify this data set; this is optional, if an automatically generated token will be returned if none is given

```r
# create a reusable, serialized ZeroMQ object with the common data on the master
w$set_common_data(fun, const, export, rettype, common_seed, token)
```

Workers that connect to the master will send a list with a field `token`. This can be used to check if the worker already received the common data it is supposed to work on.

```
if (msg$token != <token>)
    w$send_common_data()
```

Iterated data

If the worker has already received the common data, we can send it a chunk of iterated arguments to work on. These are passed as a list of iterables, e.g. a `data.frame` with a column for each iterated argument.

It also needs to have a column with name `<space>id<space>`, which will be used to identify each call.

```
chunk = data.frame(arg1=1:5, arg2=5:1, ` id `=1:5)
w$send_job_data(chunk)
```

If the worker has finished processing, it will send a message with the field `result` that is a list, containing:

- `result` - a named rettype with results
- `warnings` - a list with warning messages of individual calls
- `errors` - a list with error messages of individual calls

```
msg = w$receive_data()
if (!is.null(msg$result)) {
    # store result here, handle errors/warnings if required
}
```

Custom calls

Apart from sending common and iterated data that the worker will process in chunks, it is also possible to send arbitrary calls that it will evaluate. It needs the following fields:

- `expr` - the expression to be evaluated
- `env` - list with all additional objects required to perform the call
- `ref` - an identifier for the call; will default to the expression itself

```
w$send_call(expr, env=list(...), ref="mycall1")
```

Main event loop

Putting the above together in an event loop, we get what is essentially implemented in `master`.

```
w = workers(3)
on.exit(w$finalize())

while (we have new work to send) {
    msg = w$receive_data()

    if (!is.null(msg$result))
        # handle result

    if (msg$token != <token>)
        w$send_common_data()
    else
        w$send_job_data(...)
}

# if proper cleanup is successful, cancel kill-on-exit
```

```r
if (w$cleanup())
    on.exit()
```

A loop of a similar structure can be used to extend `clustermq`. As an example, this was done by `drake` using common data and custom calls only (no iterated chunks).

ZeroMQ message specification

Communication between the `master` (main event loop) and workers (`QSys` base class) is organised in *messages*. These are chunks of serialized data with an `id` field, and other data that is required for this type of message.

Messages per type

Below, the message `id` is listed with the additional fields per message.

Worker

This workflow is handled by the `worker()` event loop of `clustermq` (not to be confused with the `workers()` control). It is the function called in every job or thread to interact with the `master()`. The event loop is internal, i.e. it is not modifiable and not exported.

`WORKER_UP`

- Message ID indicating worker is accepting data
- Field has to be `worker_id` to master or empty to ssh_proxy
- Answer is serialized common data (`fun`, `const`, and `seed`) or `redirect` (with URL where worker can get data)

`WORKER_READY`

- Message ID indicating worker is accepting chunks
- It may contain the field `result` with a finished chunk
- If processing failed, `result` is an object of type `error`
- If success, `result` is a list with the following vectors:
  - `result` is a named `rettype` with results
  - `warnings` is a list with warning messages of individual calls
  - `errors` is a list with error messages of individual calls

`WORKER_DONE`

- Message ID indicating worker is shutting down
- Worker will send this in response to `WORKER_STOP`
- Field has to be `time` (from `Sys.time()`), `mem` (max memory used) and `calls` (number of processed calls)

`WORKER_ERROR`

- Some error occurred in processing flow (not the function calls themselves)
- Field `msg` is describing the error
- Master will shut down after receiving this signal

Master

This workflow is handled by the `master()` function of `clustermq`. If you are using `Q()` or `Q_rows()`, this is handled under the hood. Workers created outside of these functions can be reused within `Q()`/`Q_rows()` without knowing any of the internal message structure.

```
w = workers(n_jobs, ...)
# w$cleanup() for a clean shutdown at the end
```

The documentation below is to show it is possible to implement a custom control flow, e.g. if you want to evaluate arbitrary expressions on workers instead of defining one function to call and different arguments.

DO_SETUP

- Message contains common data, like the function to call and its arguments
- Required fields are: `fun`, `const`, `export`, `rettype`, `common_seed`, `token`
- Worker will respond with `WORKER_READY`

```
# create a reusable, serialized ZeroMQ object with the common data on the master
w$set_common_data()
# send this object to a worker
w$send_common_data()
```

DO_CHUNK

- Chunk of iterated arguments for the worker
- Field has to be `chunk`, a `data.frame` where each row is a call and columns are arguments
- Row names of `chunk` are used as call IDs

```
w$send_job_data()
```

DO_CALL (new in 0.8.5)

- Evaluate a specific expression on the worker
- Needs fields `expr` (the expression to be evaluated) and `env` (list environment to evaluate it in)

```
w$send_call()
```

WORKER_WAIT

- Instruct the worker to wait `wait` seconds
- Worker will respond with `WORKER_READY`

```
w$send_wait()
```

WORKER_STOP

- Instruct the worker to exit its main event loop
- This message has no fields

```
w$send_shutdown_worker()
```

Disconnect and reset socket state

```
w$disconnect_worker()
```

Control flow stages

The convention here is

- worker > master
  - master > worker

Batch processing, no proxy

This is the default use case for `Q`, `Q_rows`. It will set the common data (`DO_SETUP`; function to call, constant arguments, exported data, random seed) once and then provide chunks of arguments (`DO_CHUNK`) as `data.frames` for batch processing.

- `WORKER_UP`
  - `DO_SETUP`
- `WORKER_READY` *[repeat]*
  - `DO_CHUNK` *[repeat]*
- `WORKER_READY`
  - `WORKER_STOP`
- `WORKER_DONE`

These can be implemented the following way:

```
w$set_common_data(...)

while(work remaining or w$workers_running > 0) {
    msg = w$receive_data() # wait until a worker is ready
    if (msg$id == "WORKER_UP") { # treat same as WORKER_READY if no common data
        w$send_common_data()
    } else if (msg$id == "WORKER_READY") {
        if (work remaining)
            w$send_job_data(data.frame with arguments for all calls of this chunk)
        else
            w$send_shutdown_worker()
        # ..handle your result..
    } else if (msg$id == "WORKER_DONE") {
        w$disconnect_worker()
    } else if (msg$id == "WORKER_ERROR") {
        stop("processing error")
    }
}
```

Evaluating custom expressions

This can be mixed with batch processing, as long as `DO_SETUP` is called before `DO_CHUNK` (otherwise it will cause `WORKER_ERROR` on token mismatch).

- `WORKER_UP`
  - `DO_SETUP` or `DO_CALL` (e.g. to export commonly used data)
- `WORKER_READY` *[repeat]*
  - `DO_CALL` *[repeat]*
- `WORKER_READY`
  - `WORKER_STOP`
- `WORKER_DONE`

These can be implemented the following way:

```
w$set_common_data(...) # optional, if common data required

while(work remaining or w$workers_running > 0) {
    msg = w$receive_data() # wait until a worker is ready
    if (msg$id == "WORKER_UP") { # treat same as WORKER_READY if no common data
        w$send_common_data()
    } else if (msg$id == "WORKER_READY") {
        if (work remaining)
```

```
            w$send_call(expr, env)
        else
            w$send_shutdown_worker()
        # ..handle your result..
    } else if (msg$id == "WORKER_DONE") {
        w$disconnect_worker()
    } else if (msg$id == "WORKER_ERROR") {
        stop("processing error")
    }
}
```

Appendix: Scheduler templates

LSF

In your `~/.Rprofile` on your computing cluster, set the following options:

```
options(
    clustermq.scheduler = "lsf",
    clustermq.template = "/path/to/file/below"
)
```

The option `clustermq.template` should point to a LSF template file like the one below.

```
#BSUB-J {{ job_name }}[1-{{ n_jobs }}]  # name of the job / array jobs
#BSUB-o {{ log_file | /dev/null }}      # stdout + stderr; %I for array index
#BSUB-M {{ memory | 4096 }}             # Memory requirements in Mbytes
#BSUB-R rusage[mem={{ memory | 4096 }}] # Memory requirements in Mbytes
##BSUB-q default                        # name of the queue (uncomment)
##BSUB-W {{ walltime | 6:00 }}          # walltime (uncomment)

ulimit -v $(( 1024 * {{ memory | 4096 }} ))
CMQ_AUTH={{ auth }} R --no-save --no-restore -e 'clustermq:::worker("{{ master }}")'
```

In this file, `#BSUB-*` defines command-line arguments to the `bsub` program.

- Memory: defined by `BSUB-M` and `BSUB-R`. Check your local setup if the memory values supplied are MiB or KiB, default is `4096` if not requesting memory when calling `Q()`
- Queue: `BSUB-q default`. Use the queue with name *default*. This will most likely not exist on your system, so choose the right name (or comment out this line with an additional `#`)
- Walltime: `BSUB-W {{ walltime }}`. Set the maximum time a job is allowed to run before being killed. The default here is to disable this line. If you enable it, enter a fixed value or pass the `walltime` argument to each function call. The way it is written, it will use 6 hours if no arguemnt is given.
- For other options, see the LSF documentation and add them via `#BSUB-*` (where `*` represents the argument)
- Do not change the identifiers in curly braces (`{{ ... }}`), as they are used to fill in the right variables

Once this is done, the package will use your settings and no longer warn you of the missing options.

SGE

In your `~/.Rprofile` on your computing cluster, set the following options:

```
options(
    clustermq.scheduler = "sge",
    clustermq.template = "/path/to/file/below"
)
```

The option `clustermq.template` should point to a SGE template file like the one below.

```
#$ -N {{ job_name }}               # job name
#$ -q default                      # submit to queue named "default"
#$ -j y                            # combine stdout/error in one file
#$ -o {{ log_file | /dev/null }}   # output file
#$ -cwd                            # use pwd as work dir
#$ -V                              # use environment variable
#$ -t 1-{{ n_jobs }}               # submit jobs as array

ulimit -v $(( 1024 * {{ memory | 4096 }} ))
CMQ_AUTH={{ auth }} R --no-save --no-restore -e 'clustermq:::worker("{{ master }}")'
```

In this file, `#$-*` defines command-line arguments to the `qsub` program.

- Queue: `$ -q default`. Use the queue with name *default*. This will most likely not exist on your system, so choose the right name (or comment out this line with an additional `#`)
- For other options, see the SGE documentation. Do not change the identifiers in curly braces (`{{ ... }}`), as they are used to fill in the right variables.

Once this is done, the package will use your settings and no longer warn you of the missing options.

SLURM

In your `~/.Rprofile` on your computing cluster, set the following options:

```
options(
    clustermq.scheduler = "slurm",
    clustermq.template = "/path/to/file/below"
)
```

The option `clustermq.template` should point to a SLURM template file like the one below.

```
#!/bin/sh
#SBATCH --job-name={{ job_name }}
#SBATCH --partition=default
#SBATCH --output={{ log_file | /dev/null }} # you can add .%a for array index
#SBATCH --error={{ log_file | /dev/null }}
#SBATCH --mem-per-cpu={{ memory | 4096 }}
#SBATCH --array=1-{{ n_jobs }}

ulimit -v $(( 1024 * {{ memory | 4096 }} ))
CMQ_AUTH={{ auth }} R --no-save --no-restore -e 'clustermq:::worker("{{ master }}")'
```

In this file, `#SBATCH` defines command-line arguments to the `sbatch` program.

- Queue: `SBATCH --partition default`. Use the queue with name *default*. This will most likely not exist on your system, so choose the right name (or comment out this line with an additional `#`)
- For other options, see the SLURM documentation. Do not change the identifiers in curly braces (`{{ ... }}`), as they are used to fill in the right variables.

Once this is done, the package will use your settings and no longer warn you of the missing options.

PBS

In your `~/.Rprofile` on your computing cluster, use the SGE scheduler with a PBS template:

```
options(
    clustermq.scheduler = "sge",
    clustermq.template.lsf = "/path/to/file/below"
)
```

The option `clustermq.template` should point to a PBS template file like the one below.

```
#PBS -N {{ job_name }}
#PBS -l nodes={{ n_jobs }}:ppn={{ cores | 1 }}
#PBS -l walltime={{ walltime | 1:00:00 }}
#PBS -q default
#PBS -o {{ log_file | /dev/null }}
#PBS -j oe

ulimit -v $(( 1024 * {{ memory | 4096 }} ))
CMQ_AUTH={{ auth }} R --no-save --no-restore -e 'clustermq:::worker("{{ master }}")'
```

In this file, `#PBS-*` defines command-line arguments to the `qsub` program.

- Queue: `#PBS-q default`. Use the queue with name *default*. This will most likely not exist on your system, so choose the right name (or comment out this line with an additional `#`)
- For other options, see the PBS documentation. Do not change the identifiers in curly braces (`{{ ... }}`), as they are used to fill in the right variables.

Once this is done, the package will use your settings and no longer warn you of the missing options.

Torque

In your `~/.Rprofile` on your computing cluster, use the SGE scheduler with a Torque template:

```r
options(
    clustermq.scheduler = "sge",
    clustermq.template.lsf = "/path/to/file/below"
)
```

The option `clustermq.template` should point to a Torque template file like the one below.

```bash
#PBS -N {{ job_name }}
#PBS -l nodes={{ n_jobs }}:ppn=1,walltime={{ walltime | 30:00 }}
#PBS -o {{ log_file | /dev/null }}
#PBS -q default
#PBS -j oe

ulimit -v $(( 1024 * {{ memory | 4096 }} ))
CMQ_AUTH={{ auth }} R --no-save --no-restore -e 'clustermq:::worker("{{ master }}")'
```

In this file, `#PBS-*` defines command-line arguments to the `qsub` program.

- Queue: `#PBS -q default`. Use the queue with name *default*. This will most likely not exist on your system, so choose the right name (or comment out this line with an additional `#`)
- For other options, see the Torque documentation. Do not change the identifiers in curly braces (`{{ ... }}`), as they are used to fill in the right variables.

Once this is done, the package will use your settings and no longer warn you of the missing options.
